# Making good on a call to expand method choice for young people - Turning rhetoric into reality for addressing Sustainable Development Goal Three

**DOI:** 10.1186/s12978-017-0313-6

**Published:** 2017-04-11

**Authors:** Fariyal Fatma Fikree, Catharine Lane, Callie Simon, Gwyn Hainsworth, Patricia MacDonald

**Affiliations:** 1 Evidence to Action, PATH, 1250 23rd Street, Suite 475, Washington DC, 20037 USA; 2 GH/PRH/SDI, 2100 Crystal Drive, Crystal City, VA USA; 3 Pathfinder International, 1819 24th Ave Unit C, Seattle, WA 98122 USA; 4 Global Development, Bill & Melinda Gates Foundation, 440 5th Ave N, Seattle, WA 98109 USA; 5 CP-3 11038B1300, Pennsylvania Ave, NW, Washington, DC, 20523 USA

**Keywords:** Young people, Full contraceptive access, Full contraceptive choice, Long-acting reversible contraceptives, Call to action

## Abstract

**Background:**

Investments in the nearly two billion young people, aged 10–24 years, in the world today are necessary to meet global development commitments, specifically the Sustainable Development Goals and Ending Preventable Child and Maternal Deaths. More than 12 million married and unmarried adolescents (aged 15–19) will give birth in 2016. Complications of pregnancy and childbirth are the second leading cause of death among 15–19 year-old women and early childbearing can significantly curtail social and economic prospects for young women. Facilitating the ability of sexually active young people to choose and effectively use a satisfactory contraceptive method will ensure they can exercise their right to prevent, delay or space pregnancy.

The Global Consensus Statement, “*Expanding Contraceptive Choice for Adolescents and Youth to Include Long Acting and Reversible Contraception”* provides evidence on the safety and effectiveness of LARCs for young people. Three inter-dependent actions linking advocacy and policy (advocating for policy and guideline revisions); supply (improving quality and accessibility of an expanded method choice) and an enabling environment (social norms and comprehensive reproductive health information) are suggested as vital to achieving full access and full choice for all sexually active young people. Identified approaches include national advocacy addressing policy guidelines and standard operating procedures that guide providers in the provision of age and developmentally appropriate contraceptive services; pre-service and in-service training for health care providers to be able to effectively communicate and counsel young people, including dispelling myths and misconceptions around LARCs; and partnering with young people to design appropriate, contextually-relevant, and effective strategies to increase their self-efficacy and, at the community level, address broader social norms to dispel stigma and discrimination.

**Conclusion:**

An immediate call to action for collaborative and coordinated global, regional and national efforts that enable full access and full choice for all young people is paramount to achieve their reproductive health intentions and the Sustainable Development Goal targets.

## Background

Making good investments in the nearly two billion young people, aged 10–24 years, in the world today is paramount to the world’s ability to meet its global development commitments, specifically the Sustainable Development Goals, especially Goal Three; the United Nations Global Strategy for Women’s, Children’s and Adolescents’ Health; Family Planning 2020 (FP2020); and the United States Agency for International Development’s Ending Preventable Child and Maternal Deaths priority. This large cohort of young people who are now – or soon will be – sexually active will have a profound impact on the ability of governments to ensure the health and wellbeing of their current and future populations. Greater attention to age and developmentally appropriate information and services across the health care spectrum can mitigate the potential cost of not taking action.

Facilitating the ability of sexually active young people to choose and effectively use a satisfactory contraceptive method will ensure they can exercise their right to prevent, delay or space pregnancy. With increasing global awareness of and attention to adolescent and youth health, it is an opportune time to both establish and accelerate the implementation of policies and programs that enable young people’s full access to the widest range of contraceptive choices, including highly effective, long-acting and reversible contraception. We must stop politely and patiently waiting for change and instead must both clearly advocate for and provide guidance to global, regional, and national efforts that will allow all young people to make choices that are right for their own life circumstances and will help them realize their reproductive health aspirations.

Countless speeches, statements and declarations have called for the increased attention to the health and rights of young people through better access to sexual and reproductive health information and services, yet comprehensive action has been inadequate and fragmented. As a result poor adolescent and youth reproductive health outcomes and the attendant social consequences persist:

- Twenty eight percent of young women in developing regions are married before age 18 and 7% of girls marry before they are 15 [1].
- More than 12 million married and unmarried adolescents (aged 15–19) will give birth in 2016 [1].
- Rapid repeat pregnancy is increasingly associated with increased maternal and newborn morbidity, as well as abortions, including unsafe abortions. Reported rates of rapid, repeat pregnancy among adolescents range from 20 to 50% [2–5]. In 2015 alone, more than four million adolescents in 56 countries that ever received FP assistance from United States Agency for International Development have had a second or third child as a result of rapid repeat pregnancy [6].
- Fifty percent of unsafe abortions in the Africa region are to women under the age of 25 [7].
- Complications of pregnancy and childbirth adversely affect adolescents [8]. In 2015, maternal conditions were the leading cause of death among 15–19-year-old girls; with the highest rate (35.7 per 100,000 population) from African low and middle income countries [9]. Maternal morbidities such as fistula remain problems among adolescents [10]. In addition, early childbearing can significantly curtail social and economic prospects for young women.
- Babies born to adolescent mothers face higher odds of neonatal and infant mortality, prematurity/pre-term, stunting, underweight, diarrhea and anemia than those born to older women [11, 12].

While much work is still needed to meet the contraceptive needs of sexually active women globally, contraceptive prevalence almost doubled in the world between 1970 and 2015, from 36% in 1970 to 64% in 2015 [13], and at the same time, the level of unmet need for family planning among married or in-union women is estimated to have declined, from 22% in 1970 to 12% in 2015 [13]. However, these efforts have virtually ignored young people, especially those living in developing regions. Contraceptive use among young people, especially adolescents aged 15–19, remains despairingly low. Twenty-three million adolescents in developing regions have an unmet need for modern contraception [1]. Too often young women lack information and hold misconceptions about contraceptive methods, especially long-acting reversible contraceptives (LARCs). They may not feel supported by their partners or communities to use contraception. Many young contraceptive users experience dissatisfaction with their method or demonstrate poor method compliance (i.e., incorrect and inconsistent method use and method discontinuation). This may be, in part, due to the fact that 84% of young women aged 15–19 use less effective short-acting methods [1] which require greater effort to use correctly, require ongoing resupply, and are not always available due to stock outs.

To date, only limited efforts have been made to introduce LARCs into the method mix offered to young people. Research and practical experience reveal that one of the biggest barriers to expanding access to LARCs are providers, who are reluctant to counsel on and provide LARCs to sexually active young people [14]. Yet recent research in the United States and Kenya suggests that when young women are offered and accept the option of a LARC, they report greater method compliance and satisfaction and are less likely to experience an unintended pregnancy [15, 16]. Increasing young women’s access to the full range of contraceptive methods, including LARCs, and improving their ability to prevent unintended pregnancies holds significant promise for accelerating efforts to improve sexual and reproductive health (SRH) for young people.

## Full contraceptive access, full contraceptive choice for young people

In October of 2015, a Global Consensus Statement “*Expanding Contraceptive Choice for Adolescents and Youth to Include Long Acting and Reversible Contraception*” was released. Formally endorsed by youth organizations, professional associations, donors, and individuals, the statement provides evidence on the safety and effectiveness of LARCs for adolescents and youth and addresses commonly held myths and misperceptions regarding their effect on young women’s health and fertility. Currently hosted on the FP2020 website, (http://www.familyplanning2020.org/youth-larc-statement), the statement was well received at its formal launch at the 2016 International Conference on Family Planning. There is now a global window of opportunity for decisive, comprehensive, and collaborative action. The drafters and supporters of the Global Consensus Statement are committed to ensuring that the statement goes beyond lofty rhetoric to galvanize dramatic changes in sexual and reproductive health programs and contraceptive services for sexually active young people that include greater access to LARCs. Positioning the needs of young people within broader global efforts to increase access to a full range of contraceptive options, including LARCs, such as the guaranteed pricing of implants or the roll out of postpartum family planning including immediate postpartum intrauterine device and implant insertion, can be instrumental in accelerating progress toward young people’s full access to full method choice.

### From words to action

Numerous barriers to adolescents’ and youths access to a full range of contraceptive methods including LARCs have been documented. These include limited knowledge of their contraceptive options, myths and misconceptions, provider bias, lack of family, partner, and community support, social norms that value early pregnancy and high fertility, stigma and discrimination and an absence of youth-friendly LARCs services in facilities where many adolescents and youth access contraceptives [17–23]. In addition, national laws and policies also restrict adolescents’ and youth’s access to LARCs, or only support the use of LARCs after a first birth [17, 24–26]. We therefore identified three interrelated actions as essential to incorporating the Global Consensus Statement’s recommendations into national policies and programs. With significant overlap among these actions, their implementation should be coordinated, and collaboration among many partners is essential to achieve maximum impact. The three actions are:

1. Advocating for changes in policy, regulations and guidelines to enable young people to access comprehensive SRH services and a full range of contraceptive methods, including LARCs [17, 24–26].
2. Improving the quality and accessibility of public and private-sector contraceptive services and ensuring the provision of a full range of modern contraceptive methods, including LARCs, for all sexually active young persons [17–21].
3. Fostering an enabling environment that is free of stigma and discrimination; and ensuring young people have the information and support they need to make an informed choice about if and when to have a child, and which contraceptive method to use [22, 23].

We encourage the international non-governmental organizations that have endorsed the Global Consensus Statement to convene stakeholder meetings in the countries where they work to strategize on how best to implement these three actions, which are described in further detail below.

### Advocacy for policy change

In many countries, advocacy is needed to create a favorable policy environment that fully supports young people’s health and wellbeing, including their ability to use the contraceptive method of their choice. Countries’ pledges to FP2020 provide an excellent platform to pick up the mantle of the global consensus statement and develop, negotiate and monitor actions that increase access to LARCs for young people at country level. Further, new funding mechanisms, such as the Global Financing Facility, provide important opportunities to mobilize domestic and international resources in support of increasing young people’s access to a full range of contraceptive methods. National advocacy should address the multiple legal, socio-economic, cultural and gender related barriers that limit young people’s access to a full range of contraceptive options, including LARCs, while highlighting the health and social benefits of contraceptive use, including LARC use, by young people. Policy guidelines and standard operating procedures must be developed and/or revised to guide providers in the provision of age and developmentally appropriate contraceptive services that include access to LARCs regardless of marital status, parity and parental/partner consent. Furthermore, advocacy efforts should also push for age disaggregated data collection to help monitor progress toward ensuring young people’s access to the full range of contraceptive methods and increase accountability at country level.

Studies in the United States [15], Kenya [16] and Ethiopia (Fikree FF, Abshiro WK, Mai MM, Hagos KL and Asnake M: A simple twist to strengthening youth friendly health services!! Making the case for expanding method choice for all young Ethiopians, submitted) have demonstrated the acceptability of LARCs among sexually active young women even for delaying first birth but this is not sufficient. Advocacy efforts, at both global and national levels, could be further bolstered by additional research in other developing countries beyond Kenya and Ethiopia that assess acceptability of LARCs amongst young people, continuation of contraceptive use, and reductions in adolescent pregnancy. In addition, careful documentation of program implementation and service delivery strengthening efforts to maximize quality and choice within an implementation science paradigm will accelerate progress toward achieving full access, full choice for all young people.

### Improving the quality and accessibility of services

The World Health Organization has identified accessibility, acceptability, equity, appropriateness and effectiveness of service delivery as being essential to meeting the SRH needs and rights of young people. Further, services should be tailored to the specific socio-cultural context and needs of adolescents and youth. Few governments however have implemented “youth friendly” services at scale. Therefore, in many contexts, service delivery remains limited to stand alone clinics and boutique programs that reach few sexually active young people. There is a clear need for donors, governments, professional associations and implementing partners to work together to strengthen the capacity of health systems to provide affordable contraceptive services for young people. Attention should also be placed on ensuring that young people can access the method of their choice, including LARCs, as part of postpartum and postabortion care.

A key intervention is adequate pre-service and in-service training for health care providers to be able to effectively communicate and counsel young people, including dispelling myths and misconceptions around LARCs, and to offer a full range of contraceptive methods, including LARCs. However, simply training providers is insufficient. We must also address provider attitudes and biases around offering contraception including LARCs. Effective interventions that counter provider bias, reduce judgmental attitudes and create conducive atmospheres of privacy, confidentiality and respect are needed so that young people are empowered to access services. Strategies for supportive supervision and the collection of age disaggregated data by method are also needed to monitor improvements in contraceptive service provision to young people. Finally, programs need to collect population-based data that illustrates how increased access to and uptake of LARCs can positively influence reproductive health outcomes among sexually active young women.

### Fostering an enabling environment

Beyond unsupportive policy environments and limited access to comprehensive and quality contraceptive services, young people also face barriers to contraceptive choice at the individual level (e.g., limited sexual and reproductive health knowledge or low levels of self-efficacy) and at the community level (e.g. social norms that demand that young women prove their fertility immediately after marriage, or publicly held misconceptions that LARCs may negatively impact return to fertility). Addressing these barriers using strategies that are tailored to the diverse needs of young people are essential to ensure young people have the information and support they need to fulfill their right to choose if and when to have a child, and select from a full range of contraceptive methods.

Partnering with young people to design appropriate, contextually-relevant, and effective strategies to increase their comprehensive SRH knowledge and self-efficacy is critical. Ensuring young people have a wider choice of contraceptive options can enable them to delay the first birth and achieve their aspirations in school and beyond. Promoting school-based comprehensive sexuality education for girls in school is an excellent opportunity for sharing evidence-based information on contraceptive choices that young women can use safely and effectively. However, large numbers of young people remain outside of formal school systems making creative, community-based interventions to increase knowledge, autonomy, and self-efficacy essential to ensuring young people have *full access and full choice.*

To shift harmful and restrictive social norms that limit young people’s choice, we must work with young people to engage communities, including providers whose own biases reflect the values of the communities where they live, in reflection and dialogue on commonly held values and beliefs around adolescents, sexuality, gender, and fertility. Further, clarifying LARCs’ safety, effectiveness, and immediate return to fertility for young people can help to build consensus around offering a full array of contraceptive services, including LARCs, to young people.

## Conclusion

With the Global Consensus Statement, we have the evidence, donor support, country interest, and full cooperation of professional associations and 45 non-governmental organizations to move toward full access to full method choice for young people around the world. This commentary has laid out action steps for expanding young people’s method choice to include LARCs. We conclude with a broader question to the development community at large: *What are we waiting for?* The time for being polite and patient is long past, and we must take advantage RIGHT NOW of the window of opportunity for global, regional and national action that will enable all young people to achieve their reproductive health intentions and future aspirations.

## Abbreviations

FP: Family planning
LARC: Long-acting reversible contraceptive
SRH: Sexual and reproductive health
